# Laparoscopic Fundoplication Is Effective Treatment for Patients with Gastroesophageal Reflux and Absent Esophageal Contractility

**DOI:** 10.1007/s11605-021-05006-0

**Published:** 2021-04-26

**Authors:** Steven Tran, Ronan Gray, Feruza Kholmurodova, Sarah K. Thompson, Jennifer C. Myers, Tim Bright, Tanya Irvine, David I. Watson

**Affiliations:** 1 Department of Surgery, Flinders Medical Centre, Bedford Park, SA Australia; 2 Discipline of Surgery, Flinders University, Bedford Park, SA Australia; 3 Department of Surgery, The Queen Elizabeth Hospital, Woodville, SA Australia

**Keywords:** Fundoplication, Esophagus, Reflux, Absent contractility

## Abstract

**Background:**

Anti-reflux surgery in the setting of preoperative esophageal dysmotility is contentious due to fear of persistent long-term dysphagia, particularly in individuals with an aperistaltic esophagus (absent esophageal contractility). This study determined the long-term postoperative outcomes following fundoplication in patients with absent esophageal contractility versus normal motility.

**Methods:**

A prospective database was used to identify all (40) patients with absent esophageal contractility who subsequently underwent fundoplication (36 anterior partial, 4 Nissen). Cases were propensity matched based on age, gender, and fundoplication type with another 708 patients who all had normal motility. Groups were assessed using prospective symptom assessment questionnaires to assess heartburn, dysphagia for solids and liquids, regurgitation, and satisfaction with surgery, and outcomes were compared.

**Results:**

Across follow-up to 10 years, no significant differences were found between the two groups for any of the assessed postoperative symptoms. Multivariate analysis found that patients with absent contractility had worse preoperative dysphagia (adjusted mean difference 1.09, *p* = 0.048), but postoperatively there were no significant differences in dysphagia scores at 5- and 10-year follow-up. No differences in overall patient satisfaction were identified across the follow-up period.

**Conclusion:**

Laparoscopic partial fundoplication in patients with absent esophageal contractility achieves acceptable symptom control without significantly worse dysphagia compared with patients with normal contractility. Patients with absent contractility should still be considered for surgery.

## Introduction

Laparoscopic anti-reflux surgery is an established treatment option for the management of refractory gastroesophageal reflux disease (GERD)^1^–^3^. However, a subgroup of patients with refractory GERD exist who have concurrent esophageal dysmotility, and offering surgical management of GERD in the setting of preoperative esophageal dysmotility is contentious. Postoperative dysphagia is common in the period immediately after fundoplication, occurring in 40–70% of patients. However, it generally resolves within 3 months^4^, ^5^. Patients with preoperative dysmotility are considered by many to be at significant risk of persistent dysphagia, although definitions of dysmotility are inconsistent, with the exception of a complete absence of peristalsis. The term “scleroderma esophagus” has been used previously to refer to a pattern of esophageal dysmotility characterized by absent esophageal contractility in the distal esophageal smooth muscle and reduced lower esophageal sphincter tone. This pattern of motility is not exclusive to scleroderma and is also seen in other connective tissue disorders, metabolic and endocrine disorders, and neuromuscular disorders^6^. It is also seen in some individuals presenting for anti-reflux surgery who do not have any systemic disorder^7^, ^8^.

In an attempt to mitigate the risk of persistent dysphagia following fundoplication, the original Nissen fundoplication has been modified to various partial fundoplication options such as the Dor and Toupet fundoplications, aiming to reduce the likelihood of creating an over-competent lower esophageal sphincter^9^, ^10^. It has been suggested that a partial fundoplication is a better option for individuals with motility disorders, as it might be less likely to create a gastroesophageal junction luminal pressure greater than esophageal contractile pressure. However, many patients with absent esophageal contractility are not offered or referred for surgical management by clinicians due to concerns about an excessive risk of persistent dysphagia.

A cohort of patients with absent motility who also have severe reflux symptoms despite maximal medical therapy exists. There is emerging evidence to suggest that partial fundoplication in these patients is a safe and effective means of treating their medically refractory GERD^11^–^13^. However, studies exploring the role of fundoplication in those with absent esophageal contractility are generally limited by small numbers, a lack of longer-term outcomes, and a lack of matched controls. The aim of this study was to determine the long-term outcomes after fundoplication in patients with absent esophageal contractility, compared to those with normal esophageal contractility.

## Materials and methods

A case-control study was performed using information obtained from a prospectively maintained database^12^. Preoperative, operative, and postoperative data for 2686 patients undergoing laparoscopic fundoplication was prospectively collected from December 1991 through to December 2014 and entered chronologically into a computerized database (FileMaker Pro Version 5.5, FileMaker Inc., Santa Clara, California, USA). Follow-up in this study was approved by the Southern Adelaide Health Human Research Ethics Committee. Patients underwent surgery across multiple hospitals in Adelaide, South Australia. All patients had a laparoscopic fundoplication for persistent gastroesophageal reflux symptoms refractory to proton pump inhibitors. Preoperatively, all patients underwent routine esophageal manometry studies, either conventional or high-resolution manometry (HRM) when it became available. The choice of fundoplication type was at the discretion of the operating surgeon. Early in the experience a 360° Nissen fundoplication calibrated with a 52Fr intra-esopahgea bougie, and without division of short gastric vessels was the standard antireflux procedure constructed. Subsequently, and for the majority of the reported experience, an anterior partial fundoplication was performed. Our surgical technique for anterior partial fundoplication has been described in detail previously^14^, ^15^.

Patients with absent esophageal contractility on their preoperative manometric assessment were identified from the database. Absent contractility was defined according to the Chicago classification (v4.0) as normal esophago-gastric junction relaxation and 100% failed peristalsis on esophageal manometry (HRM – distal contractile integral (DCI) < 100 mmHg.cm.s; conventional manometry – contractile amplitude < 30 mmHg)^16^, ^17^. Initial electronic screening involved restricting the database to only include patients with 100% failed peristalsis or missing data for this variable. Manometry reports for potential cases were then manually reviewed in duplicate (ST, RG) to confirm that the inclusion criteria were satisfied, and that all included patients had manometry findings consistent with absent contractility. Concurrently, patients without evidence of major or minor disorders of peristalsis (demonstrated 80–100% successful swallows) were identified as possible controls. A control group with normal esophageal motility was then formed via propensity matching in a 1:20 ratio. Matching was undertaken for age, sex, and fundoplication type. Normal motility was defined as propagation of at least 80% of water swallows with a distal peristaltic amplitude of at least 40 mmHg. Patients with manometric features of achalasia were not included in this database. Patients with other major motility disorders (diffuse esophageal spasm, hypercontractile esophagus) or minor motility disorders (ineffective or weak esophageal motility) were excluded. Patients whose original manometry data and reports were not available for review, and patients with no follow-up data available were also excluded.

Patients were followed up at 3 months following surgery, and then yearly, via a symptom assessment questionnaire. Patients were mailed the questionnaire with a reply-paid envelope. Patients who did not return the questionnaire were sent a second copy; if this was not returned, follow-up was attempted by telephone. The questionnaire assessed a number of domains on 0–10 analog scales including heartburn, dysphagia for solids and liquids, regurgitation, and satisfaction with surgery. This questionnaire has been used extensively across 3 decades and with specific scoring systems validated in previous studies^18^. For this study, a comparison of the symptom scores between the two groups was undertaken at 1, 5 and 10 years follow-up. A subgroup of patients with systemic sclerosis was identified within the patients with absent contractility. A subgroup analysis was performed comparing these symptom domains between patients with both systemic sclerosis and absent contractility to the remainder of patients with absent contractility.

Statistical analysis was performed using Stata Statistical Software: Release 16 (College Station, TX: StataCorp LLC). Data are expressed as medians and interquartile ranges, mean and standard deviation, or frequencies. Baseline comparisons between absent contractility and the control group were performed by independent *t* test, Mann-Whitney U tests, or Chi-squared tests as appropriate. To assess the significance of changes in measurement scores over multiple time points whilst taking into account the correlation from the within-individual measurements, a mixed linear model with random intercept for each individual was used. Measurements were found not to meet normality assumptions. Thus, the mixed linear model results were bootstrapped using 200 bootstrap samples. Multivariate models were adjusted for age at operation, type of fundoplication, and gender. Interactions between groups and multiple time points were included in the models and the preoperative time was used as the comparison point. Data from mixed linear models are presented as estimated marginal means (95% bootstrapped confidence intervals). The type 1 error rate was set at *p* < 0.05 for all tests of statistical significance.

## Results

### Patients

**Fig. 1:**
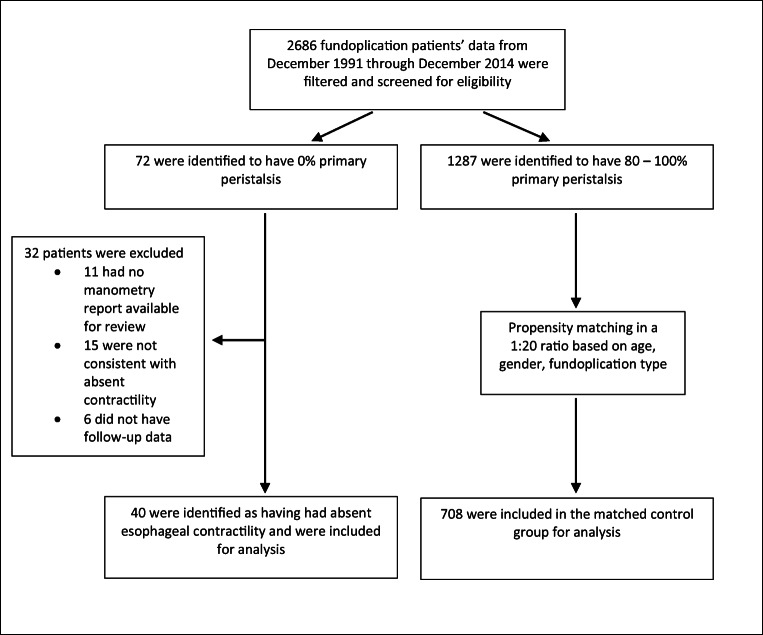
Enrolment and matching of patients who have undergone fundoplication

From December 1991 through December 2014, 40 patients were identified with absent contractility and matched to 708 controls with normal peristalsis (Fig. 1). Median follow-up time for both groups was 5 years (IQR, 2-10). The mean age at the time of operation was 52.1 years (SD 13.2) for those with absent contractility and 51.9 years (SD 13.1) for those with normal peristalsis. The baseline characteristics of both groups were similar (Table 1). Approximately equivalent ratios between males and females were obtained from propensity matching. In those with absent contractility prior to surgery, 1-year, 5-year, and 10-year follow-up for surgical outcomes were available for 36, 25, and 18 patients, respectively. For those with normal esophageal contractility, 561 had 1-year follow-up outcomes, 450 had 5-year, and 282 had 10-year outcome data. The fundoplication was successfully completed for all patients in the series.

**Table 1:** Baseline characteristics

	Absent contractility *n* = 40	Normal contractility *n* = 708
Mean age at time of operation (SD)	52.1 (13.2)	51.9 (13.1)
Gender M/F	20/20	353/355
Median follow-up time (IQR)—years	5 (2–10)	5 (2–10)
Preoperative Manometry*		
•Mean primary peristalsis %	0	95.5
•Mean LES/EGJ resting pressure mmHg	4.4	11.0
•Mean LES/EGJ nadir pressure mmHg	0.8	1.8
Total number of reoperations Indications for reoperation:	7 (17.5%)	61 (8.6%)
•Dysphagia	2	17
•Recurrent reflux	4	26
•Regurgitation	-	1
•Recurrent hiatus hernia	1	11
•Hiatal stenosis	-	4
•Malignancy	-	1
•Delayed gastric emptying	-	1
Fundoplication type		
•90° anterior (anatomical)	11	142
•180° anterior	25	327
•360° Nissen	4	239
Systemic sclerosis	7	0

*Not all patients had high resolution manometry, but all patients in conventional and high-resolution manometry groups had measures of their primary peristalsis, lower esophageal sphincter (LES) or esophagogastric junction (EGJ) resting and nadir pressures/integrated relaxation pressure.

### Heartburn

There was no statistically significant difference for heartburn scores between those with absent contractility and normal contractility preoperatively and at all follow-up intervals (Table 2). Preoperatively, patients with absent contractility had a significantly higher adjusted mean heartburn score by 1.59 (95% CI 0.29–2.89, *p* = 0.02). There were no other significant differences at other time points. Both patient groups demonstrated a sustained reduction in heartburn scores over the entire postoperative follow-up period when compared to the baseline preoperative scores—this was statistically significant at each follow-up point for both groups. There was no statistically significant difference when the change in scores between both patient groups and follow-up time were compared (Table 3).

**Table 2:** Univariate analysis of symptoms and satisfaction with surgery over time.

	Absent contractility	Normal contractility	*p* value
Preoperative			
Number available for follow-up	40	708	
Median 0-10 Heartburn Score (IQR)	10 (9–10)	9 (7–10)	0.10
Median 0-10 Dysphagia Liquid Score (IQR)	0 (0–1)	0 (0–4)	0.92
Median 0-10 Dysphagia Solid Score (IQR)	2 (0.5–4.5)	1 (0–6)	0.81
Median 0-10 Regurgitation Score (IQR)	9 (8–10)	8 (6–10)	0.30
One-year follow-up			
Number available for follow-up	36	561	
Median 0-10 Heartburn Score (IQR)	1 (0–4)	0 (0–2)	0.09
Median 0-10 Dysphagia Liquid Score (IQR)	0 (0–1.5)	0 (0–1)	0.24
Median 0-10 Dysphagia Solid Score (IQR)	2 (0–5)	1 (0–3)	0.07
Median 0-10 Regurgitation Score (IQR)	1.5 (0–6)	0 (0–2)	0.18
Median 0-10 Satisfaction with Surgery Score (IQR)	9 (7.5–10)	9 (8–10)	0.55
Five-year follow-up			
Number available for follow-up	25	450	
Median 0-10 Heartburn Score (IQR)	2 (0–4)	1 (0–3)	0.40
Median 0-10 Dysphagia Liquid Score (IQR)	0 (0–0)	0 (0–2)	0.27
Median 0-10 Dysphagia Solid Score (IQR)	0 (0–2)	1 (0–3)	0.09
Median 0-10 Regurgitation Score (IQR)	3 (0–4)	1 (0–3)	0.16
Median 0-10 Satisfaction with Surgery Score (IQR)	9 (6–10)	9 (7–10)	0.55
Ten-year follow-up			
Number available for follow-up	18	282	
Median 0-10 Heartburn Score (IQR)	2 (0–6)	1 (0–4)	0.16
Median 0-10 Dysphagia Liquid Score (IQR)	0 (0–2)	0 (0–2)	0.88
Median 0-10 Dysphagia Solid Score (IQR)	2 (0–5)	1 (0–4)	0.56
Median 0-10 Regurgitation Score (IQR)	2 (0–7)	1 (0–3)	0.48
Median 0-10 Satisfaction with Surgery Score (IQR)	9 (8–10)	9 (8–10)	0.78

**Table 3:** Multivariable regression model for each symptom

		Heartburn		Dysphagia liquid		DYSPHAGIA SOLID		REGURGITATION	
	Follow-up point – years	Adjusted mean (95% CI)	*p* value	Adjusted mean (95% CI)	*p* value	Adjusted Mean (95% CI)	P value	Adjusted Mean (95% CI)	P value
Difference in Scores Between Absent Contractility & Normal Contractility	0 (Preoperative)	1.59 (0.29, 2.89)	0.02	-0.08 (-0.78, 0.62)	0.82	1.09 (0.01, 2.17)	0.048	1.76 (-1.98, 5.49)	0.36
	1	0.66 (-0.34, 1.65)	0.20	0.57 (-0.19, 1.33)	0.14	1.32 (0.28, 2.36)	0.01	0.30 (-3.60, 4.21)	0.88
	5	0.58 (-0.61, 1.77)	0.34	-0.09 (-0.93, 0.76)	0.85	-0.21 (-1.33, 0.91)	0.71	1.91 (-1.13, 4.95)	0.22
	10	0.94 (-0.40, 2.29)	0.17	0.09 (-0.85, 1.03)	0.85	0.91 (-0.37, 2.19)	0.16	0.96 (-1.89, 3.82)	0.51
	11+	1.47 (-0.27, 3.21)	0.10	-0.20 (-1.62, 1.23)	0.79	0.54 (-0.80, 1.86)	0.43	0.61 (-2.34, 3.56)	0.69
Change in Scores for Absent Contractility	1 vs 0	-4.82 (-6.64, -3.00)	0	0.36 (-0.73, 1.46)	0.52	0.01 (-1.68, 1.71)	0.99	-6.36 (-10.82, -1.89)	0.005
	5 vs 0	-4.18 (-6.10, -2.26)	0	-0.14 (-1.00, 0.72)	0.75	-1.32 (-2.69, 0.06)	0.10	-2.01 (-6.59, 2.56)	0.39
	10 vs 0	-3.63 (-5.84, -1.43)	0.001	0.15 (-1.07, 1.37)	0.81	0.19 (-1.51, 1.90)	0.83	-1.63 (-6.86, 3.61)	0.54
	11+ vs 0	-2.81 (-5.24, -0.38)	0.02	-0.05 (-1.70, 1.59)	0.95	-0.22 (-1.89, 1.45)	0.80	-1.82 (-7.37, 3.73)	0.52
Change in Scores for Normal Contractility	1 vs 0	-3.89 (-4.24 -3.54)	0	-0.29 (-0.52, -0.06)	0.01	-0.22 (-0.52, 0.08)	0.15	-4.90 (-5.88, -3.93)	0
	5 vs 0	-3.17 (-3.56, -2.79)	0	-0.14 (-0.39, 0.11)	0.27	-0.02 (-0.33, 0.30)	0.92	-2.17 (-3.00, -1.33)	0
	10 vs 0	-2.99 (-3.39, -2.58)	0	-0.02 (-0.30, 0.26)	0.88	0.37 (0.01, 0.73)	0.05	-0.83 (-1.46, -0.21)	0.01
	11+ vs 0	-2.69 (-3.06, -2.31)	0	0.06 (-0.18, 0.30)	0.61	0.33 (0.04, 0.63)	0.03	-0.67 (-1.31, -0.03)	0.03
Difference in Change in Scores Between Absent Contractility & Normal Contractility	1 vs 0	-0.93 (-2.79, 0.92)	0.32	0.65 (-0.48, 1.78)	0.26	0.23 (-1.47, 1.93)	0.78	-1.45 (-5.97, 3.07)	0.53
	5 vs 0	-1.01 (-2.95, 0.93)	0.31	-0.001 (-0.89, 0.89)	0.998	-1.30 (-2.90, 0.30)	0.11	0.15 (-4.30, 4.61)	0.95
	10 vs 0	-0.65 (-2.86, 1.57)	0.57	0.17 (-1.08, 1.43)	0.79	-0.18 (-1.93, 1.57)	0.84	-0.79 (-5.97, 4.39)	0.76
	11+ vs 0	-0.12 (-2.56, 2.32)	0.92	-0.12 (-1.80, 1.57)	0.89	-0.55 (-2.25, 1.14)	0.52	-1.15 (-6.64, 4.34)	0.68

### Dysphagia

There were no significant differences in median dysphagia scores for both liquids and solids between those with absent contractility and normal contractility at all follow-up intervals (Table 2). No differences in mean scores for dysphagia with liquids were found upon multivariate analysis (Table 3). Over time, there was no statistically significant change in dysphagia with liquids found between the two groups. Preoperatively, patients with absent contractility had a significantly higher adjusted mean score for dysphagia with solids by 1.09 (95% CI 0.01–2.17, *p* = 0.048) compared with those with normal contractility. This trend persisted at 1-year follow-up, with a higher adjusted mean score of 1.32 (95% CI 0.28–2.36, *p* = 0.01) in patients with absent contractility compared with those with normal peristalsis. No significant differences were found at five or more years between the patient groups. For patients with absent contractility, there were no significant changes in score at follow-up, compared to the preoperative score. For patients with normal contractility, at 11+ years of follow-up, dysphagia scores for solids were 0.33 higher (95% CI 0.04–0.63, *p* = 0.03). No significant differences were found when comparing the difference in score change between the two groups.

### Regurgitation

Preoperatively there was no significant difference in median regurgitation scores between those with absent contractility and those with normal contractility. Postoperatively, there was no difference in median scores between the two groups (Table 2). In patients with absent contractility, there was a statistically significant improvement in regurgitation scores at 1-year follow-up; however, there was no difference at long-term follow-up. A similar trend was observed in those with normal contractility—at 1 year the adjusted mean score was 4.90 units lower (*p* < 0.001). This persisted at long-term follow-up, but the magnitude decreased from −4.90 to −0.67. When comparing the difference between the change in scores over time for both groups, there was no statistically significant difference found (Table 3).

### Satisfaction with surgery

Postoperatively, the vast majority of patients were satisfied with the outcomes of the surgery. Median scores in the first year for those with aperistalsis and normal peristalsis were 9/10 (IQR 7.5–10) and 9/10 (IQR 8–10), respectively. Median scores remained at 9/10 for the duration of follow-up. There were no statistically significant differences in median scores for satisfaction with surgery between the groups at 1 year, 5 years and 10 years (Table 2). Multivariate analysis demonstrated that scores for satisfaction with surgery were on average higher in males by 0.42 compared with females (*p* = 0.001) (Table 4).

**Table 4:** Multivariable regression model for satisfaction with surgery

	Adjusted mean	95% Confidence interval	*p* value
Wrap Type			
360° Nissen (reference)	-	-	-
180° anterior	-0.02	-0.31, 0.28	0.92
90° anterior	-0.46	-0.82, -0.10	0.01
Gender			
Male	0.42	0.16, 0.68	0.001
Female (reference)	-	-	-
Age at Operation	0.008	-0.002, 0.017	0.10
Follow-up Point	-0.02	-0.04, -0.01	0.001
Absent Contractility	0.05	-0.55, 0.56	0.99
Follow-up & Absent Contractility	-0.04	-0.11, 0.03	0.27

### Systemic sclerosis patients

Seven of the 40 patients in the absent contractility group had systemic sclerosis and were compared with the other 33 patients in the absent contractility group. Preoperatively, patients with systemic sclerosis appear to be comparable to the rest of the patients with absent contractility. No differences were seen between median scores for any of the symptom domains. Postoperatively, there were no differences in median scores for any of the outcomes at the first follow-up assessment (Table 5).

**Table 5:** Subgroup analysis comparing patients with systemic sclerosis and absent contractility vs patients without systemic sclerosis

	Absent contractility		
	Systemic sclerosis	No systemic sclerosis	*p* value
Preoperative			
Number available for follow-up	7	33	
Median Heartburn Score (IQR)	10 (10–10)	10 (8.5–10)	0.49
Median Dysphagia Liquid Score (IQR)	0 (0–0)	0 (0–1)	0.45
Median Dysphagia Solid Score (IQR)	4 (4–4)	1 (0–5)	0.51
Median Regurgitation Score (IQR)	9 (9–9)	8.5 (8–10)	0.79
First visit follow-up			
Number available for follow-up			
Median Heartburn Score (IQR)	2 (0–4)	1.5 (1–4)	0.94
Median Dysphagia Liquid Score (IQR)	0.5 (0–1)	0 (0–2)	0.80
Median Dysphagia Solid Score (IQR)	2.5 (1–6)	2 (0–6)	0.46
Median Regurgitation Score (IQR)	1.5 (0–3.5)	2.5 (0–4)	0.66
Median Satisfaction with Surgery Score (IQR)	8 (7–9)	9 (8–10)	0.14

## Discussion

Fundoplication is a validated treatment option for medically refractory GERD. However, concern remains about offering or undertaking fundoplication for those with disorders of esophageal motility. There is a belief that fundoplication in those with preoperative ineffective esophageal motility results in an increased risk of persistent dysphagia, particularly if a Nissen fundoplication is proposed. This has resulted in the practice of tailoring anti-reflux surgery^19^, ^20^. Some practitioners avoid offering or referring for surgery all together. Evidence continues to emerge demonstrating that a tailored approach may not be required for those with ineffective esophageal motility. Most recently, Nikolic et al. published a case-controlled series of 72 patients with ineffective esophageal motility who underwent laparoscopic Nissen Fundoplication. They found no difference in postoperative dysphagia rates, quality of life, or need for revisional surgery at a median 5-year follow-up.^13^ Similarly, Booth et al. conducted a randomized controlled trial comparing Nissen and Toupet fundoplications in those with ineffective motility and effective motility. They found that there were no differences in postoperative symptoms at 6 months and 1 year after surgery, and concluded that there was no reason to adopt a tailored approach.^21^ Strate et al. also conducted a randomized trial of 200 patients, and similarly found that postoperative dysphagia rates at 4 months and two years after surgery did not correlate with preoperative manometry findings of ineffective esophageal motility.^22^, ^23^ Another group has also reported similar findings at a mean 5.8 years after 240° and 360° fundoplication.^24^ However, these studies generally included patients who had varying levels of dysmotility and often excluded patients with severe dysmotility or absent contractility.

The clinical outcome after fundoplication is much less clear for the subset of patients with the most severe dysmotility issue – absent contractility of the esophagus. Literature describing fundoplication outcomes in those with absent contractility is limited. Studies published so far are case series, with no control groups. These studies, however, do support the idea that partial fundoplication can be safely offered to patients with reflux and an aperistaltic esophagus. Our group has previously published a case series of 26 patients describing their outcomes following fundoplication – it appeared that reasonable reflux control was able to be obtained without persistent dysphagia, although only 14 of these patients were followed beyond 2 years.^12^ Goldberg et al. described a cohort of 13 patients with absent contractility (including 10 patients with systemic sclerosis) who underwent predominantly Toupet fundoplication. At mean 36 weeks follow-up, they similarly found that laparoscopic fundoplication was acceptable in their patients with absent contractility.^11^ Most recently, Armijo et al. published a case series of 52 patients, 9 of which had absent contractility. They again found similar results for 6- and 12-month outcome, demonstrating that fundoplication is a feasible treatment option for patients with absent contractility.^25^

This current study is one of the largest cohorts of patients with absent contractility published, with a significant follow-up period. The results show that long-term postoperative outcomes for fundoplication in individuals with absent contractility appear comparable to those with normal motility. Reflux symptoms appeared to be well controlled, and the incidence of dysphagia was not increased at short and long-term follow-up. Patients with absent contractility who have undergone fundoplication also demonstrated comparable satisfaction with the surgery at follow-up. These findings are consistent with the previously published studies, further strengthening the proposition that absent contractility should not be a contraindication for surgery. Unfortunately, our series of patients with absent contractility is too small to draw conclusions about the fundoplication type that should be used. Our study included 4 patients with absent contractility who very early in our experience underwent a standard Nissen fundoplication when it was the routine operation performed in our unit, and no specific adaptations were made to the Nissen fundoplication technique applied in the patients with absent motility. The small number of patients who underwent a Nissen fundoplication, however, are not sufficient to demonstrate that a Nissen fundoplication is appropriate in this setting, and for now any conclusion that a fundoplication can be performed for patients with absent contractility should perhaps be limited to the setting of an anterior partial fundoplication.

Over the last 2 decades, our group has progressively moved away from routine use of the Nissen fundoplication in favor of a partial fundoplication, even in patients without significant motility disorders. This recommendation relates to the fact that an anterior partial 180° fundoplication has been shown to provide acceptable reflux control with a lower rate of persistent postoperative dysphagia compared to a Nissen fundoplication.^26^ Physiologically, this is explained by a reduction in the resistance imposed at the gastroesophageal junction by the fundoplication wrap.^27^ Therefore in the setting of preoperative manometry findings demonstrating absent contractility, we would advocate for a partial fundoplication.

The population of patients with systemic sclerosis is yet another subgroup where the indications for surgery are unclear. Systemic sclerosis is a connective tissue disorder with significant gastrointestinal involvement. These patients often have varying degrees of impaired esophageal contractility and concurrent GERD. Early studies published in the literature evaluating anti-reflux surgery in this population had mixed findings. Some demonstrated reasonable reflux control after fundoplication without worsening dysphagia^28^, ^29^, while others had limited success^30^. In our series, 7 patients who had absent esophageal contractility and a diagnosis of systemic sclerosis were included in the analysis. Their outcomes appear to be comparable to the other 33 patients with absent contractility, without significantly different symptom outcomes. This suggests that patients with systemic sclerosis are also able to safely undergo a fundoplication. However, definitive conclusions are unable to be drawn due to the limited number of patients.

This study has a number of strengths. This is one of the largest published series of patients with absent contractility. It is also the only study to compare patients with absent contractility against a propensity matched control group with normal contractility. Of note it was found that males were overall more satisfied with surgery compared to females. This is a finding that has previously been described in other patient cohorts undergoing anti-reflux surgery, adding to the external validity of this study.^31^

This study also has a number of limitations. Firstly, given that this is a retrospective review of a database, results are dependent on accurate entry of data. However, the data in our case was prospectively obtained and maintained. With the limited number of patients available with an aperistaltic esophagus, the conclusions that can be drawn are limited. In particular, concrete conclusions about the type of wrap to perform cannot be established and our findings should be limited to the context of an anterior partial 180° fundoplication, which was constructed in 36 of the 40 patients in the study group. Furthermore, all of our study outcomes are subjective measures and symptom outcomes, as high rates of compliance with objective measures at long-term follow-up have been difficult to achieve. Lastly, given the rarity of absent esophageal contractility in our population, it is not feasible to perform a prospective randomized trial. In an attempt to mitigate this, the large propensity matched control group provides a more robust comparison group than any previous study.

## Conclusion

Laparoscopic anti-reflux surgery in the form of an anterior partial fundoplication in patients with absent esophageal contractility achieves comparable long-term outcomes when compared to patients with normal motility. Patients with medically refractory reflux who have absent contractility should still be considered for surgical intervention.
